# l-Arginine Supplementation and Metabolism in Asthma

**DOI:** 10.3390/ph4010187

**Published:** 2011-01-12

**Authors:** Nicholas J. Kenyon, Michael Last, Jennifer M. Bratt, Vivian W. Kwan, Erin O'Roark, Angela Linderholm

**Affiliations:** Pulmonary and Critical Care Medicine, University of California Davis, 451 Health Sciences Drive, GBSF, Rm. 6517, Davis, CA 95616, USA

**Keywords:** asthma, l-arginine, nitric oxide, arginase, ADMA

## Abstract

l-Arginine, the amino acid substrate for nitric oxide synthase, has been tested as a therapeutic intervention in a variety of chronic diseases and is commonly used as a nutritional supplement. In this study, we hypothesized that a subset of moderate to severe persistent asthma patients would benefit from supplementation with l-arginine by transiently increasing nitric oxide levels, resulting in bronchodilation and a reduction in inflammation. The pilot study consisted of a 3 month randomized, double-blind, placebo-controlled trial of l-arginine (0.05 g/kg twice daily) in patients with moderate to severe asthma. We measured spirometry, exhaled breath nitric oxide, serum arginine metabolites, questionnaire scores, daily medication use and PEFR with the primary endpoint being the number of minor exacerbations at three months. Interim analysis of the 20 subjects showed no difference in the number of exacerbations, exhaled nitric oxide levels or lung function between groups, though participants in the l-arginine group had higher serum l-arginine at day 60 (2.0 ± 0.6 × 10^−3^ *vs.* 1.1 ± 0.2 × 10^−3^ μmol/L, p < 0.05), ornithine at day 30 (2.4 ± 0.9 *vs.* 1.2 ± 0.3 μmol/L serum, p < 0.05) and ADMA at day 30 (6.0 ± 1.5 × 10^−1^ *vs.* 2.6 ± 0.6 × 10^−1^ μmol/L serum, p < 0.05) on average compared to the placebo group. The study was terminated prematurely. Supplementing asthma subjects with l-arginine increases plasma levels; whether subgroups might benefit from such supplementation requires further study.

## 1. Introduction

Asthma is a disease that has brought nitric oxide (NO) chemistry and therapeutics to the forefront of basic and clinical research. As a component of exhaled breath, NO concentration is currently used as a noninvasive biomarker for eosinophilic airway inflammation and clinical exacerbations. Because of its correlation with inflammation severity, a number of clinical studies have been directed at blocking NO production based upon the presumption that NO acts to potentiate inflammation through the formation of reactive nitrogen species. More recent studies now suggest this assumption may not be accurate [1,2] and that the development of therapeutic agents to block NO production in asthma may have been premature [3].

*De novo* synthesis of NO from the substrate, l-arginine is catalyzed by the nitric oxide synthase (NOS) enzymes. As a semi-essential amino acid, l-arginine is readily available over the counter and is popular as a nutritional supplement to increase muscle mass. More recently, l-arginine has been tested as a potential therapeutic in numerous acute and chronic disease states, including sickle cell chest crisis, pulmonary artery hypertension, coronary heart disease, pre-eclampsia and myocardial infarction, because of its bronchodilator and vasodilator actions. In these trials, l-arginine was administered as a nitric oxide synthase (NOS) substrate in a vasodilatory capacity, directly infused into either the peripheral arterial or pulmonary arterial beds. In sickle cell disease patients with acute chest syndrome, a deficiency in vascular-derived NO is thought to contribute to the pulmonary vascular red cell sickling and vasoconstriction [4]. In combination with hydroxyurea, l-arginine supplementation augments the production of NO and improves outcomes in this disease [5].

In addition to the apparent similarities in NO metabolism in the pathophysiology of sickle cell disease and asthma, sickle cell disease patients have one of the highest reported co morbidity rates of asthma (40–60%) [6]. Sickle cell anemia and asthma both exhibit altered l-arginine metabolism resulting from increased competition between the arginase and nitric oxide synthase enzymes for the common substrate, l-arginine. Heightened l-arginine metabolism resulting from chronic inflammation can also lead to decreased plasma l-arginine, as detected in asthmatic patients [5]. The culmination of these concepts make the proposal of an l-arginine-based intervention in asthmatics viable as well as a potential safe treatment for asthmatics, especially individuals who do not respond to conventional therapeutics.

Results from animal model studies support the idea that NO protects against structural airway changes that are the sequelae of chronic inflammation. We have previously shown that mice with the targeted deletion of the NOS2 gene, an isoform of NOS highly expressed in asthma, develop increased airway fibrosis and airway hyperresponsiveness after chronic antigen exposure. Based on these observations in animal models and clinical studies, the question arises whether a NOS substrate like arginine would affect airways inflammation and hyperresponsiveness, improving asthma care.

In this study, we hypothesized that a therapeutic intervention that augments NO production, such as oral l-arginine supplementation, would decrease asthma exacerbations and improve the care of a subset of severe asthma patients, with the potential to dramatically alter the care of some “difficult-to-control” asthma patients. We specifically investigated the central hypothesis that NO generated in the airways of more severe asthmatics decreases exacerbations, asthma symptoms, airway inflammation, and airway hyperresponsiveness. To this end, we performed a randomized, double-blinded, placebo-controlled trial in moderate persistent asthmatics to determine whether three months of oral l-arginine supplementation would decrease asthma exacerbations and airways hyperresponsiveness.

## 2. Experimental

### 2.1. Study Design

We performed a three month randomized, double-blind, placebo-controlled, parallel group trial approved by the UC Davis Human Subjects Institutional Review Board and the UC Davis Clinical and Translational Science Center (CTSC) review committee. The study was registered with the clinicaltrials.gov website (NCT00280683) and was performed at the UC Davis CTSC Clinical Research Center at the Mather Veterans Administration Hospital, Sacramento, CA, USA.

### 2.2. Participants

Moderate to severe persistent asthma patients, as defined by the NAEPP Expert Panel Reports, were eligible for enrollment [10]. Most subjects were recruited from the UC Davis Asthma Network clinics, which are referral clinics for patients with difficult to control asthma [11]. Eligible subjects had documented moderate to severe persistent asthma, were at least 18 years of age, not pregnant, and able to give consent. Subjects did not have an acute exacerbation at the time of enrollment and were on the same asthma medications for at least one month. The exclusion criteria included a history of intubation in the past year, pregnancy, proven lack of adherence to controller asthma therapy, a baseline FEV1% < 40% predicted, known or suspected allergy to l-arginine, current smokers or subjects with more than 15 pack-year history of smoking. All subjects were on standard controller therapy including appropriate doses of inhaled corticosteroids and long-acting bronchodilators, and had an asthma action plan. At the time of enrollment, subjects had a full medical history and physical examination performed, their spirometry measured, a St. George's Respiratory Questionnaire performed (SGRQ), and a sample of exhaled breath gas collected. The SGRQ was chosen because of its validation in both the severe asthma and chronic obstructive lung disease patient population.

### 2.3. l-Arginine Intervention

The randomization process and disbursement of l-arginine (0.05 g/kg twice daily; 6–10 g/day) and placebo were done by the UC Davis Investigational Drug Service to ensure that both the physician and participant were blinded. The subjects began the study medication on day 0 and continued for 90 days and were asked to discontinue use of any nutritional supplements prior to the start of the study. l-arginine is a naturally occurring amino acid that is available as a supplement at grocery stores and health food stores without a prescription. As a nutritional supplement, it is not regulated by the Food and Drug Administration and is readily available for public consumption.

The initial formulation of the l-arginine was a solution; l-arginine powder was dissolved in orange-flavored syrup. After three weeks, it became apparent that the l-arginine in syrup was being converted to ammonia. We subsequently purchased 1 gram l-arginine tablets and these were disbursed, along with the placebo tablets, by the Investigational Drug Service. The documented side effects of the l-arginine were minimal and included dyspepsia 10%, diarrhea 3%, and bad taste 5%. Given that this study had little associated risk and potentially good benefits for those individuals, the risk benefit ratio of this study was favorable.

Follow-up visits occurred at approximately days 30 and 60 with the final visit at day 90. At each visit, an interim history and examination, a review of the participant's symptom diary, a blood draw, spirometry, an SGRQ questionnaire, and exhaled breath gas sample was performed. Between visits, participants measured their peak expiratory flow rate on a home monitor on a daily basis and they recorded the dosage and frequency of rescue albuterol and prednisone use or changes. Adherence to study regimen was monitored by scheduled telephone call between visits and pill counting when prescription containers were returned. Dietary l-arginine was not assessed.

### 2.4. Data Collection

The primary endpoint of the study was number of exacerbations in three months. Exacerbations were defined as any of the following [12-14]: (a) a drop in AM peak expiratory flow rate (PEF) >30% from baseline on two consecutive days; (b) need for initiation of or increased dose of inhaled corticosteroids; (c) doubling of short-acting β-agonist use (e.g. albuterol)/ day. The secondary clinical endpoints that were measured included the patient symptom diary, daily short acting β-agonist use, St. George's Respiratory questionnaire, daily PEF diary, forced expiratory volume in one second (FEV1), and forced expiratory volume in one second to forced vital capacity ratio (FEV1/FVC) measurements performed at the monthly visits, exhaled NO concentration and induced sputum eosinophil percent counts.

### 2.5. Exhaled NO and Serum NOx Measurement

To collect exhaled breath gas to measure exhaled NO, subjects inspired clean filtered air through the mouthpiece with a Rudolph valve (Sievers Corp) to a full lung volume. Once at full volume, they expired at a slow flow rate of 0.35 L/sec into a Mylar bag which was then sampled for NO with a Sievers NO Analyzer (Sievers) within 3 h of the collection [2]. Each participant repeated the collection twice and exhaled NO samples were taken on study days 1, 45, 75, and 105. Serum nitrate and nitrite in the previously isolated blood samples were measured as an indicator of NO production through reduction with acidified vanadium III using the Sievers NO Analyzer (Sievers, Boulder, CO, USA).

### 2.6. Measurement of l-Arginine, l-Arginine Metabolites, ADMA and Polyamines in Serum Samples

The downstream product of arginase, l-ornithine, was measured in the serum each month and compared to the amount of l-citrulline, the downstream product of the competing NOS enzymes. Aliquots of 100 uL of serum sample were deproteinized using acetone (4:1 [vol/vol] acetone: serum). The resulting supernatant was removed and evaporated to dryness using a vacuum centrifuge. The samples were then analyzed for basic amino acid content and ADMA by reverse-phase HPLC after pre-column derivatization with phenylisothiocyanate (PITC) (15) or polyamines *via* pre-column derivatization with dansyl chloride.

### 2.7. Sample Size and Statistical Analysis of Data

The sample size calculation was performed using the open-source statistical program ‘R’ (2004), and based on a desired significance level of 0.05 and a β error = 0.2; the desired N for each group was 17. A total of 40 subjects were planned, and an interim analysis was performed after subject number 20 completed the study. In regards to performing our planned analyses and correlations, we were faced with an interesting statistical problem. With a small sample size, most of the standard assumptions underneath conventional statistical analysis, such as normality of errors, could not be assumed to hold. In order to correct for the multiple hypothesis tests we planned, we rejected the Family-Wise Error Rate, such as the Bonferroni Correction, as overly conservative and instead, chose to control the False-Discovery Rate (FDR) [18]. There are many methods to control the FDR [19], but we restricted ourselves to that of Benjamini and Hochberg [18] allowing a higher power for rejecting our second and subsequent hypotheses, at a cost of a less strong conclusion.

For parametric analysis of data, a *t*-test with the appropriate degrees of freedom was used. Linear regression analysis was performed by a weighted least squares method. Correction for multiple comparisons and, where appropriate, unequal standard deviations between groups, was performed. For non-parametric analysis, a Wilcoxin test was used. Patient-specific effects were estimated and ranked for each effect of interest, using a standard ANOVA model. The effects were ranked, and a Wilcoxon rank-sum test was conducted comparing the two groups of interest. *P*-values were then computed, and significance declared based on a FDR of 0.1.

## 3. Results and Discussion

Twenty subjects were enrolled in the study and fifteen completed the three month trial. The most common reasons for subjects discontinuing the study included inability to adhere to the study visit schedule and/or protocol. All subjects had moderate or severe persistent asthma as diagnosed by an asthma specialist, and most were recruited from a cohort of patients followed by the UC Davis Asthma Network (UCAN™) [11]. All subjects were adherent to their medication regimen of inhaled corticosteroids and long-acting bronchodilators. Twenty percent of the patients were being administered anti-IgE therapy (omalizumab™) bimonthly. After 20 subjects or one-half of the planned cohort was enrolled in this pilot study, an interim analysis was performed blindly by an outside investigator to determine if there was any affect of l-arginine on asthma exacerbation rates. No difference was found in this primary endpoint of asthma exacerbations in the three month time period and the study was discontinued and the preliminary data were analyzed.

### 3.1. Patient Data

There were two males and eight females in each group. Baseline data and medications for each group are listed in Table 1a and Table 2. There were no significant differences in age, baseline FEV1%, baseline FVC%, or peak expiratory flow rate (PEFR) between the placebo and l-arginine group. The baseline expiratory NO concentration (FeNO) for the placebo group was 22.4 ± 5.5 and for the l-arginine group was 28.2 ± 5.8. The baseline SGRQ scores for the placebo group and the l-arginine group respectively were 40.4 ± 7.0 and 38.9 ± 4.9. Of this cohort, eight subjects in the l-arginine group and seven subjects in the placebo group completed the study. One subject in the placebo group was unblinded and discontinued from the study because of an unexplained increase in systolic blood pressure >200 mmHg. One subject in the l-arginine group was discontinued because of unremitting headache after 60 days, and one other subject stopped for personal reasons.

### 3.2. Exhaled NO and Serum NOx Measurements

**Figure 1:**
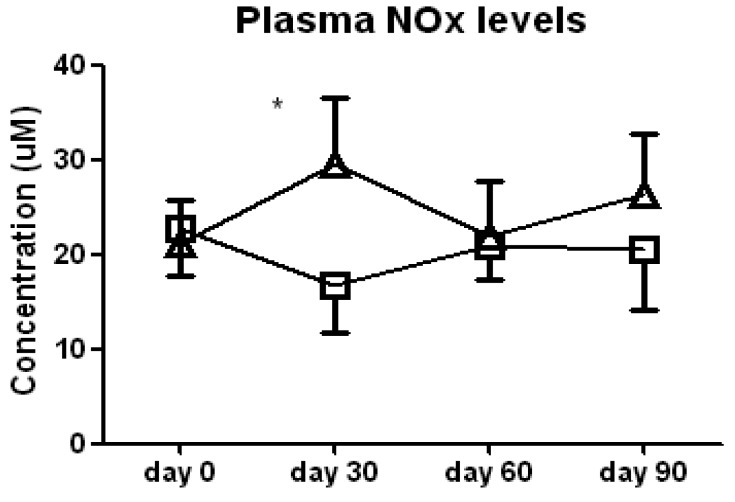
Serum nitrate and nitrite (NOx) concentration in subjects treated with l-arginine (triangles) or placebo (squares). Data are presented as mean ± SEM. * denotes *p* < 0.05.

We found no significant difference between the l-arginine and placebo groups in either FeNO levels or serum nitrate/nitrite (NOx) levels when all of the data were analyzed by two-way ANOVA, indicating an absence of a sustained increase in NO. However, *t*-tests of the data at each study visit noted a significant increase in serum NOx in the l-arginine treated patients compared to the placebo group at day 30 of the study (29.6 ± 9.6 *vs.* 16.6 ± 5.1 μM, *p* < 0.05, Figure 1). This difference was due to both a decrease in NOx in the placebo group and a concomitant increase in the l-arginine treated group. This suggests that any increase in NO in the lung or systemically that occurs with the supplementation with l-arginine is transient, and probably reflects compensatory mechanisms. Improvement in clinical symptoms does not appear to correlate with a sustained increase in NO in this cohort of more severe asthmatics, therefore NO may not be a good airway inflammation surrogate marker in this subset of patients.

### 3.3. Asthma Exacerbations

We found no difference in the total number of minor exacerbations summed over three months between the two groups of patients. Overall, the total numbers of exacerbations were low in both groups. Our original power analysis was based on an expected minor exacerbation rate of 3–4 per month, as defined above. Instead, our subjects demonstrated much better asthma control than expected. The summed total number of exacerbation in the l-arginine group, was 30 (4 ± 1) and in the placebo group, 31 (4 ± 1). Extending our study to the full cohort of 40 subjects would not have shown a significant difference between groups for this primary endpoint.

### 3.4. Lung Function

We performed a correlation analysis between FEV1 values and group and found no differences between them. Other secondary endpoints, including daily peak flow rate, cough, and dyspnea scores, were also compared between the two treatment groups and no significant differences were found. While certain patients who received l-arginine did have a significant increase in PEF rates after the initiation of l-arginine therapy that correlated with an increase in FeNO levels, this was not consistent. Further serum or exhaled condensate marker profiles, or genotypes, are needed to better understand possible responders to l-arginine chronically.

### 3.5. Arginine Metabolites

**Figure 2:**
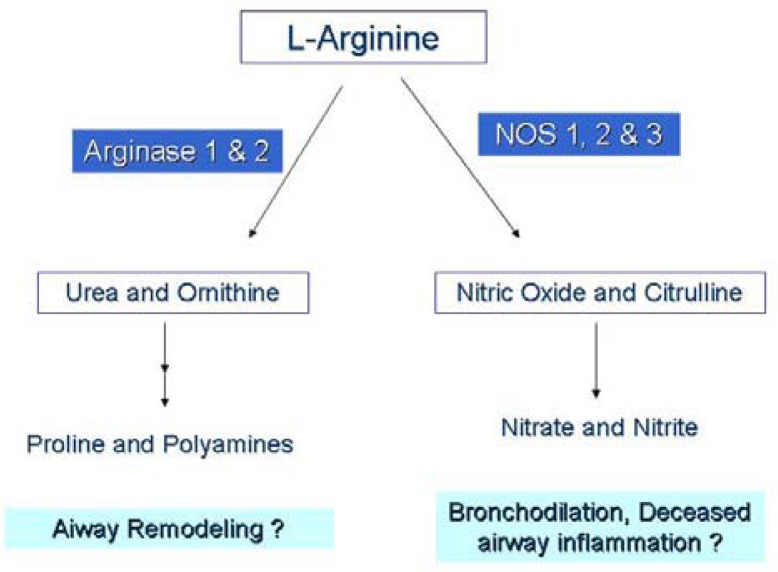
Diagram depicting the major pathways and downstream metabolites of l-arginine. Nitric oxide synthase enzymes (NOS 1, 2, 3) may be in direct competition with arginase for the substrate l-arginine, thereby affecting nitric oxide production.

**Figure 3:**
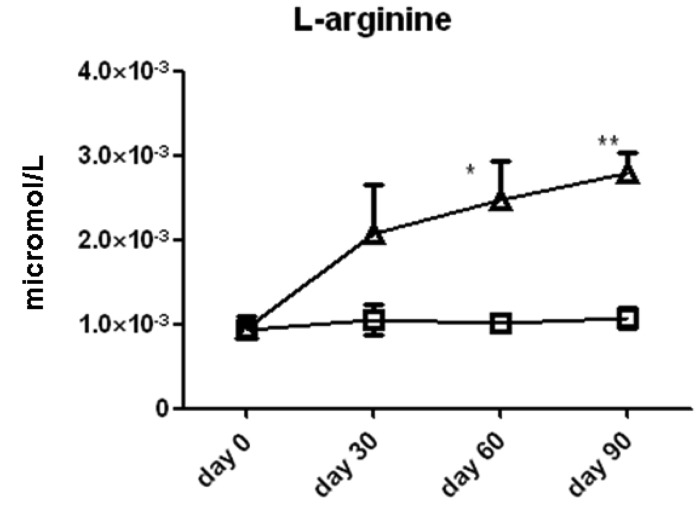
l-Arginine content per 100 μL of serum in subjects treated with l-arginine (triangles) or placebo (squares). Data are presented as mean ± SEM. * denotes *p* < 0.05 and ** denotes *p* < 0.01.

We measured the serum concentrations of l-arginine and its downstream metabolic products via the arginase (ornithine, proline, spermine, spermidine, cadaverine, and putrescine) and NOS (citrulline) enzyme pathways (Figure 2). Time series analysis with two-way ANOVA showed that serum l-arginine concentrations increased significantly in the l-arginine intervention group compared to the placebo group. l-arginine concentration was significantly higher in the l-arginine treated group compared to the placebo group at the 60 day (2.0 ± 0.6 × 10^−3^ *vs.* 1.1 ± 0.2 × 10^−3^ μmol/L, *p* < 0.05) and 90 day (2.4 ± 0.5 × 10^−3^ *vs.* 1.0 ± 0.1 × 10^−3^ μmol/L, *p* < 0.01, Figure 3) time points, when analyzed by individual *t*-tests.

**Figure 4:**
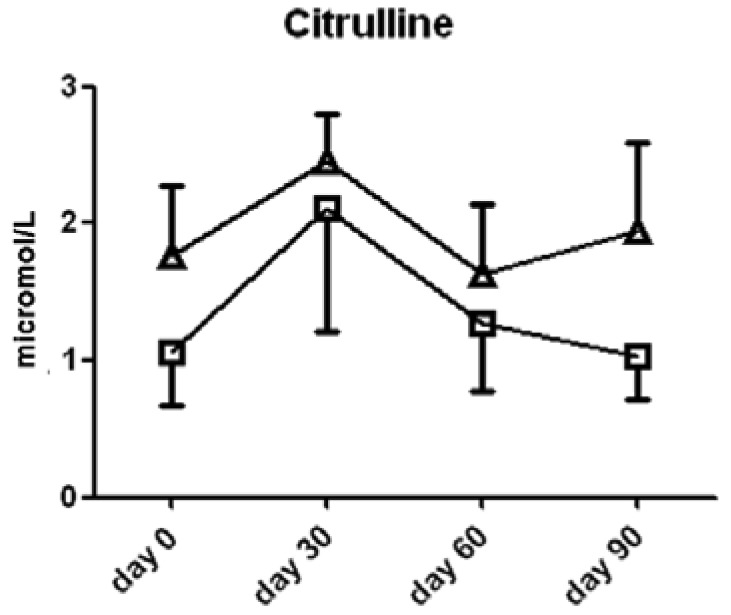
Citrulline content in serum from subjects treated with l-arginine (triangles) or placebo (squares). Data are presented as mean ± SEM.

Interestingly, l-citrulline, a product of the NOS reaction with l-arginine, was not significantly different between the two groups of subjects (Figure 4).

**Figure 5:**
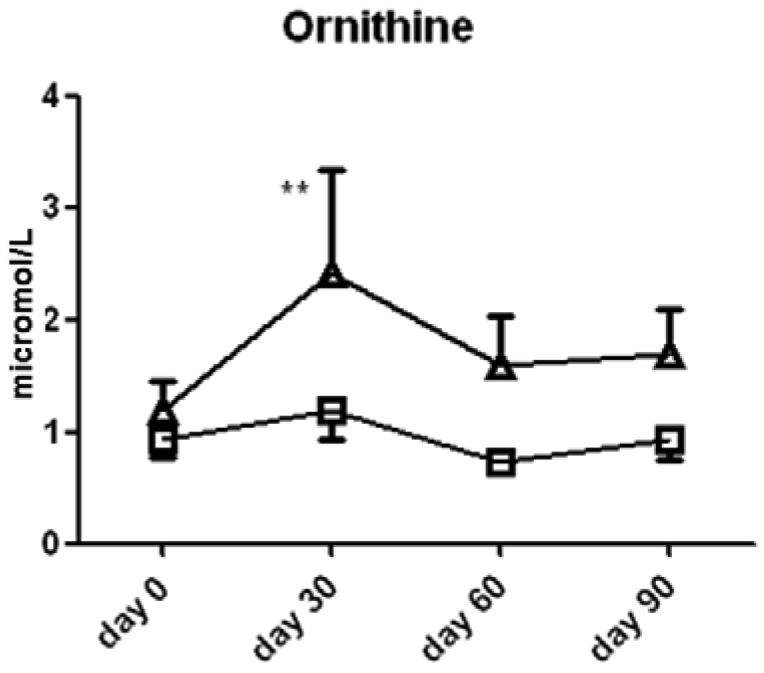
Serum ornithine content in serum from subjects treated with l-arginine (triangles) or placebo (squares). Data are presented as mean ± SEM. * denotes *p* < 0.05.

**Figure 6:**
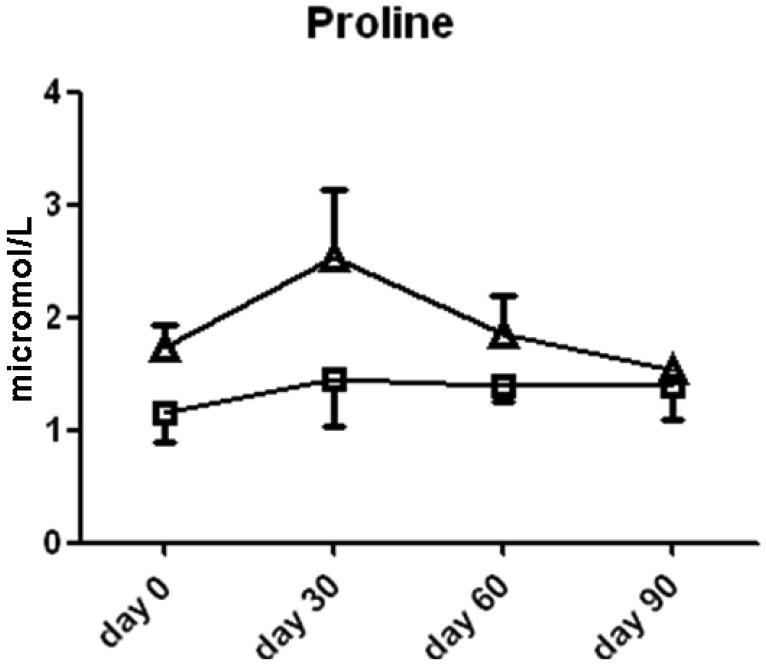
Proline content in serum from subjects treated with l-arginine (triangles) or placebo (squares). Data are presented as mean ± SEM.

Ornithine content was significantly higher in the l-arginine treated group compared to the placebo group at the 30 day (2.4 ± 0.9 *vs.* 1.2 ± 0.3 μmol/L serum, *p* < 0.05, Figure 5) time point, thereby suggesting that the majority of the l-arginine substrate may have been metabolized by the arginase enzymes which are present in both the lung, liver, kidney and red blood cells. The cellular source of arginase activity can not be addressed fully in this study. Proline content was not different between the two groups (Figure 6).

**Figure 7:**
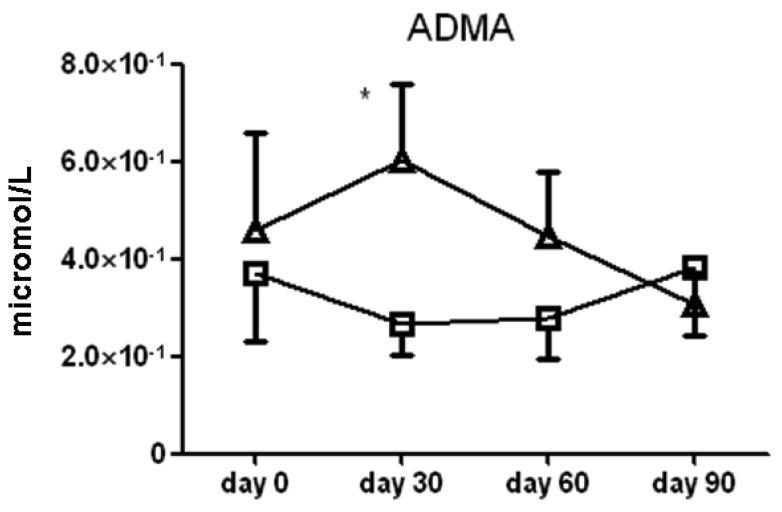
Serum asymmetric dimethyl arginine (ADMA) content from subjects treated with l-arginine (triangles) or placebo (squares). Mean ± SEM. * denotes *p* < 0.05.

There were no significant differences between l-arginine and placebo treated cohorts among any of the small molecule polyamines. Of note, serum asymmetric dimethylarginine (ADMA) content was significantly higher in the l-arginine treated group compared to the placebo group at the 30 day (6.0 ± 1.5 × 10^−1^ *vs.* 2.6 ± 0.6 × 10^−1^ μmol/L serum, *p* < 0.05) time point only (Figure 7). Another analysis showed that there was no significant difference seen in spermidine, one of the polyamines measured, amongst the two treatment groups (data not shown). Finally, there was large patient to patient variability in the putrescine, cadaverine and spermine data, and we found no significant differences between l-arginine and placebo treated cohorts in any of these assays.

### 3.7. Treatment

There is renewed interest in nitric oxide as both an exhaled breath biomarker and a therapeutic target in patients with persistent asthma. While the development of therapeutic compounds to augment or inhibit NO generation continues, many basic questions about NO chemistry and function in the airway remain unanswered. It is not known what the appropriate concentrations of NO should be in an asthmatic patient either at baseline or with an infectious exacerbation; or how changes in exhaled NO concentrations reflect changes in total NO pools in the various compartments of the lung.

In this study, l-arginine was administered orally, thus the plasma l-arginine concentration at any given time point depended on the dynamic interconversion of metabolites across multiple organ systems. Absorption of l-arginine occurs in the small intestine, converting l-arginine first to ornithine and from ornithine to citrulline using the arginase (ARG) and ornithine transcarbamylase (OTC) enzymes, respectively. Citrulline can be reconverted to l-arginine in the kidney. l-Arginine and its downstream metabolites, ornithine and citrulline, can be also be derived from the degradation of proteins. Asymmetric dimethylarginine (ADMA), another arginine metabolite, is derived predominantly from protein degradation.

The concentration of free l-arginine in the plasma is dependent upon the complex balance of endogenous synthesis and nutritional intake with cellular uptake and body-wide catabolic metabolism. As a result, local fluctuations in l-arginine concentration are eventually distributed throughout the body's organs, making tissue or organ specific changes in concentration difficult to detect. Despite these limitations, decreases in plasma l-arginine have been detected in asthmatic patients [5]. This may result from either a combined effect of increased arginase activity in the lung itself, or increase arginase activity in the serum.

The arginase and nitric oxide synthase enzymes both utilize l-arginine as a substrate and concomitant expression of these enzymes in inflamed tissues, specifically the Arg1 and NOS2 isoforms, has led to speculation that the depletion of l-arginine by arginase reduces NO production by the NOS enzymes. While the kinetics of the two competing enzymes would otherwise discredit this theory, this discrepancy can be explained by cellular compartmentalization of the enzymes [21] creating unequal concentration gradients or substrate pools [22,23]. Heightened arginase activity can create fluctuations in localized tissue l-arginine concentrations [5] and studies in macrophages have observed that the NOS2 isoform is dependent upon the extracellular l-arginine pool when intracellular l-arginine depletion occurs [24-26]. In asthma, it is suggested that the arginase and NOS enzymes are in competition for the substrate l-arginine at the level of the airway compartment [27,28].

In this study, the dosage of l-arginine used (6–8 g/day) is conservative compared to doses used in previous clinical studies, (3–30 g/day [19-23]), but represents a significant increase from the daily dietary intake of between 4–5 g/day based on an average daily diet in the U.S. [29]. In our pilot study, the 6–8 g/day dose of l-arginine did not decrease the number of minor exacerbations in moderate to severe asthmatic subjects, but a subset of the subjects that received l-arginine appeared to show clinical improvement. This observation lends credence to the idea that a subset of asthmatic patients on regular controller therapies may benefit from this therapeutic approach.

We expected that any rise in exhaled NO concentration would be transient, as numerous compensatory factors regulate production over time. Overall, we observed no difference in exhaled NO levels between the l-arginine and placebo groups. With our monthly sampling regiment, it is likely that we missed NO fluctuations early in the regimen. Personal NO monitors that would allow study participants to measure home readings several times per day may be needed to properly record shorter timescale shifts in metabolism.

Of the metabolites measured, supplemental l-arginine increased serum ornithine levels most consistently. This suggests that substrate loading of NOS does not necessarily result in increased NO production. Both arginase and NOS are induced in the airways of asthmatics [30] and ovalbumin-exposed mice [31,32] and our results suggest that arginase I and/or II may be the dominant enzymes in the airways of moderate to severe asthmatics regulating NO.

Rodent disease models, including hepatic ischemia-reperfusion injury [33,34], endothelial dysfunction [35,36], and Leishmania infection [25,37], have shown that modulation of the l-arginine metabolic pathways by arginase inhibition or l-arginine supplementation can be an effective treatment. Studies examining the mouse model of allergen-induced airway inflammation also collectively confirm that supplementing mice with l-arginine or arginase inhibitors [32] can manipulate the arginase-NOS pathway. Despite variability in outcome depending on the intervention method used and protocol, these treatment methods can lead to changes in lung tissue l-arginine concentrations, as well as physiological changes in the lungs including airway inflammation and airway hyperresponsiveness. Given the relative safety profile of l-arginine, it afforded the opportunity to translate findings in mice directly to humans. We were able to manipulate the l-arginine concentration in plasma, and although no measurable improvement in lung parameters was observed between the two groups, a subset of the supplementation group claimed to have a subjective improvement in their quality of life. A strategy that modulates arginase expression or activity perhaps in combination with l-arginine substrate loading for NOS may be more efficacious.

There is significant interest in the methylated forms of arginine metabolites in asthma and other diseases, specifically ADMA, because it acts as an inhibitor of the NOS enzymes and can affect NO production and can uncouple the NOS enzymes resulting in the production of superoxide. Other factors that can increase serum ADMA levels include oxidative stress, which can alter ADMA metabolism by decreasing the activity of dimethylarginine dimethylaminohydrolase (DDAH), the enzyme that converts ADMA to l-citrulline and dimethylamine. Previous cardiovascular trials have suggested that ADMA may lead to increased adverse vascular events, and studies are now focusing on direct modulation of this compound [38].

In a recent severe asthma consortium study, there were few differences in arginine metabolites among a healthy control, mild asthmatic, and severe asthmatic subject population [39] but there were higher serum concentrations of ADMA in the severe asthma cohort compared to the other two. In our study, we observed an increase in ADMA levels of approximately two-fold at the 30-day time point. ADMA did not continue to increase or accumulate after the first month, and we did not note any significant adverse events related to this increase, but developing strategies to counteract the effects of ADMA may also prove to be important for l-arginine or NO-enhancing treatments.

### 3.9. Study Limitations

Further studies should consider determining the subset of subjects likely to respond to this therapy by either metabolic screening of exhaled breath condensate or serum, or searching for specific NOS and arginase gene polymorphisms that may affect enzyme activity or expression. The parallel group study design proved challenging for subject recruitment as these asthma subjects were on numerous asthma medications and were uninterested in adding three months of placebo. A cross-over design trial may have improved subject recruitment and retention. The slow rate of recruitment in this pilot-funded study led to delays in sample processing for some early subjects, and ultimately triggered our decision to perform the blinded interim analysis.

Pharmacogenetic screening to calculate benefit/risk ratio based on NOS/arginase gene polymorphism patterns in the subject pool was not feasible in this study as it would require a very large number of subjects and the data were not available to support this analysis. Matching of global gene profiles with supplemental arginine response patterns may allow us to find patient subsets that would better respond to l-arginine supplementation.

The use of the false discovery rate (FDR) in our analysis of the data depends on the hypothesis being independent. If the dependencies were sparse, an independence assumption is an acceptable simplification of the model. The methods for controlling the family-wise error rate do not have this shortcoming, since the error rate is based on never wrongly rejecting a null hypothesis [40].

## 4. Conclusions

The renewed development of NO donating compounds may show benefit in patients with asthma not responding to medical therapy. In this small pilot study, l-arginine supplementation to augment NO levels and affect NO function did not improve the lung function or symptoms of a general cohort of moderate to severe asthmatics. It remains to be seen whether a subgroup of responders to l-arginine therapy exists. The possibility that a subset of such patients could improve with this readily available therapy remains intriguing, however. Subsequent studies with supplemental l-arginine should focus on whether the increases in serum ornithine and proline downstream of arginase lead to excessive collagen deposition in the lung and airway remodeling. Further clinical studies should focus consider determining the subset of subjects likely to respond to this therapy by either global metabolomic screening or outlining specific NOS and arginase gene polymorphisms that appear to confer benefit.

## Figures and Tables

**Table 1:** Baseline study participants (values are expressed as mean ± SD).

**Subject Characteristics**	**Placebo Subjects**	**l-arginine Subjects**	**P value**
N	10	10	-
Mean age, y	50.8 ± 15.1	53.1 ± 15.4	0.75
Sex, male/ female	2/8	2/8	-
Exhaled NO, ppb	24.4 ± 17.6	28.2 ± 16.4	0.64
FEV1, L	2.24 ± 0.93	2.10 ± 0.67	0.71
FEV1 % predicted	70.8 ± 18.5	75 ± 17.5	0.63
FVC, L	3.04 ± 1.25	2.80 ± 0.6	0.63
FVC% predicted	79.8 ± 19.9	85.2 ± 14.6	0.52
FEV1/FVC ratio (%)	74 ± 11	73 ± 10	0.85
PEFR, L/min	315 ± 106	310 ± 70	0.89
SGRQ questionnaire score	40.4 ± 22.2	38.9 ± 13.7	0.87

**Table 2:** Baseline study participant asthma medications at study enrollment.

**Medications**	**Placebo Subjects (% taking medication)**	**l-arginine Subjects (% taking medication)**
Inhaled steroids	100	100
Inhaled long-acting β2-agonists	100	100
Oral steroids	60	40
Anti IgE (omalizumab)	20	20
Leukotriene receptor antagonists	80	80
